# Attachment, Symptom Severity, and Depression in Medically Unexplained Musculoskeletal Pain and Osteoarthritis: A Cross-Sectional Study

**DOI:** 10.1371/journal.pone.0119052

**Published:** 2015-03-25

**Authors:** Corinna Schroeter, Johannes C. Ehrenthal, Martina Giulini, Eva Neubauer, Simone Gantz, Dorothee Amelung, Doreen Balke, Marcus Schiltenwolf

**Affiliations:** 1 Department of Orthopedics, Trauma Surgery and Paraplegiology, Heidelberg University Hospital, Schlierbacher Landstr. 200a, 69118 Heidelberg, Germany; 2 Department of General Internal Medicine and Psychosomatics, Heidelberg University Hospital, Thibautstr. 2, 69115 Heidelberg, Germany; 3 SRH Gesundheitszentrum Bad Wimpfen, Bei der alten Saline 2, 74206 Bad Wimpfen, Germany; University of Texas Health Science Center at San Antonio, Research Imaging Institute, UNITED STATES

## Abstract

**Background:**

Attachment insecurity relates to the onset and course of chronic pain via dysfunctional reactions to pain. However, few studies have investigated the proportion of insecure attachment styles in different pain conditions, and results regarding associations between attachment, pain severity, and disability in chronic pain are inconsistent. This study aims to clarify the relationships between insecure attachment and occurrence or severity of chronic pain with and without clearly defined organic cause. To detect potential differences in the importance of global and romantic attachment representations, we included both concepts in our study.

**Methods:**

85 patients with medically unexplained musculoskeletal pain (UMP) and 89 patients with joint pain from osteoarthritis (OA) completed self-report measures of global and romantic attachment, pain intensity, physical functioning, and depression.

**Results:**

Patients reporting global insecure attachment representations were more likely to suffer from medically unexplained musculoskeletal pain (OR 3.4), compared to securely attached patients. Romantic attachment did not differ between pain conditions. Pain intensity was associated with romantic attachment anxiety, and this relationship was more pronounced in the OA group compared to the UMP group. Both global and romantic attachment anxiety predicted depression, accounting for 15% and 17% of the variance, respectively. Disability was independent from attachment patterns.

**Conclusions:**

Our results indicate that global insecure attachment is associated with the experience of medically unexplained musculoskeletal pain, but not with osteoarthritis. In contrast, insecure attachment patterns seem to be linked to pain intensity and pain-related depression in unexplained musculoskeletal pain and in osteoarthritis. These findings suggest that relationship-informed focused treatment strategies may alleviate pain severity and psychological distress in chronic pain independent of underlying pathology.

## Introduction

### Psychosocial factors in chronic pain

The experience of chronic pain is multifactorial, and neither merely biologically determined nor a purely psychogenic phenomenon. Current models conceptualize chronic pain as resulting from complex interactions between biological and psychosocial factors [1]. Psychosocial influences may function as a dispositional vulnerability for the development of chronic pain or affect its maintenance and progression. For example, personal characteristics such as beliefs about the meaning of bodily sensations or about self-efficacy affect the way patients perceive or cope with pain, and thereby influence the course of pain-related disease. Anxious patients, who hypervigilantly monitor even the smallest signals from their body, may avoid exercise out of fear of aggravating their symptoms. In contrast, other patients may be hyposensitive to bodily signals due to developmental experiences demanding perseverance in the face of severe life stress [2].

Chronic pain may or may not have an underlying organic cause in terms of a medically identifiable pathology. However, in both cases, physiological and psychological changes will occur as a consequence [2]. These complex and interindividually varying interactions make it difficult to disentangle psychosocial from physical factors in the development and occurrence of pain conditions, and complicate treatment approaches. Current clinical intervention strategies still fail to help a significant proportion of patients. Therefore, matching treatment to patient subgroups based on psychosocial characteristics is considered important [3]. Our study seeks to disentangle the psychosocial vulnerability factor *attachment* from other, especially organic factors by identifying attachment patterns and associations between attachment and symptom severity in two different groups of patients with musculoskeletal pain: One with a clearly defined organic cause (osteoarthritis, OA) and one without (medically unexplained musculoskeletal pain, UMP).

### Internal working models of attachment

Adult attachment theory provides a developmental framework for interindividual differences in cognitive, emotional, and behavioral responses to pain. Insecure adult attachment patterns are considered a risk factor for the development of chronic pain, as well as poor prognosis in the face of chronic pain [4]. According to attachment theory, humans have a biologically determined need for proximity [5], particularly in the face of stressors such as pain. As a result of repeated interactional experiences with primary caregivers, internal working models of attachment start to develop. Internal working models are schemata that organize cognition, emotion, and behavior in relationships throughout the lifespan [6], which are crucial for the development of affect regulation, and stress resilience [7–9]. On a cognitive level, they comprise a “model of self,” which determines the extent to which individuals consider themselves worthy of support and proximity, and a “model of others,” which affects individuals’ confidence in receiving support from others [10]. The combination of these models of the self and other yields a classification of one secure and three insecure attachment styles: dismissing, preoccupied, and fearful [11]. On a regulatory level, working models also determine individual strategies of hyperactivation (“attachment anxiety”) or deactivation (“attachment avoidance”) of attachment-related information processing [7].

Internal working models have been conceptualized in recent studies as hierarchically organized neural network structures, running from memories of particular relationship partners, through representations of different relationship domains (i.e., romantic partners, family, and friends), to central tendencies, which form a context-sensitive, generic attachment working model [12]. Because general and relation-specific attachment patterns are correlated, but not necessarily identical, the outcome of an attachment measurement depends on the level of relationship representation it addresses. Specifically, general working models from the highest level of abstraction are not only relevant for primary attachment relationships, but can also color interactions with non-attachment figures such as health care providers [13]. For example, global insecure attachment styles have been linked with enhanced or reduced health care utilization [14]. In contrast, more specific romantic relationship models may determine whether the relationship with a romantic partner serves as a source of social support or of distress. The relevance of marital interaction patterns on the progression and severity of psychosomatic symptoms, even on their daily fluctuation, has been demonstrated in numerous studies [15–17]. However, most studies assessed either global or romantic attachment styles, complicating the interpretation of results on measurement-specificity in an unambiguous manner. Therefore, it is worthwhile to explore the importance of both levels of attachment (global and romance-specific) in health conditions such as chronic pain.

### Attachment as a psychosocial vulnerability factor

Insecure attachment is a widely acknowledged risk factor for not only psychopathology, but also adverse health behavior and outcome in a variety of health conditions [18–19]. Recent findings indicate that insecure attachment is particularly associated with impaired stress regulation [18], increased symptom reporting [14], medically unexplained symptoms [20], and somatoform disorders [21]. For example, insecure attachment styles occurred more frequently in somatoform facial pain than in trigeminal neuralgia [22]. Even insecurely attached individuals not currently suffering from chronic pain conditions show increased catastrophizing and hypervigilance, as well as decreased pain thresholds and self-efficacy to episodes of acute pain or experimentally induced pain [5, 15, 23]. These findings suggest that attachment insecurity is related to the development of chronic pain via dysfunctional reactions to episodes of acute pain [24].

However, results on direct associations between attachment patterns and pain intensity and disability among patients with chronic pain are mixed [24]: some authors reported no direct association between insecure attachment and pain intensity [25–27] or disability [26] in heterogeneous chronic pain samples, while others found a more varied picture when using homogenous subsamples and different attachment measures. For example, romantic anxious attachment was associated with pain intensity and disability in patients suffering from arthritis [28], and romantic fearful attachment was linked to pain severity in patients with lung cancer [17]. In patients suffering from chronic widespread pain (CWP), general preoccupied attachment was associated with disability and number of pain sites, but not with pain intensity [29]. The findings are more consistent with regard to the associations between attachment and psychological distress; in chronic pain patients, general insecure attachment has been linked to catastrophizing [25, 30], lower self-efficacy [26], and depression [25, 31].

In sum, insecure attachment is associated with dysfunctional reactions to acute pain and psychological distress in chronic pain. Inconsistent findings with regard to pain intensity and disability can be attributed to undetected differences between specific chronic pain conditions, or between differences in assessment of general vs. romantic attachment patterns. Based on the evidence linking attachment theory with chronic pain, Meredith [4] presented an attachment-diathesis model of chronic pain, proposing that insecure attachment represents both a vulnerability factor for the development of chronic pain as well as for poor outcome in the face of chronic pain. An examination of these relationships in pain conditions with different etiologies might thus help tailor attachment-informed intervention approaches to different subgroups and settings.

### Research questions

The present study seeks to contribute to the understanding of the potentially differential impact of attachment as a dispositional vulnerability factor for the occurrence of chronic pain conditions, or as a factor affecting their progression and severity. To address these questions, we systematically compared two different pain subgroups, one with (severe osteoarthritis, OA), and one without (medically unexplained musculoskeletal pain, UMP) a clearly defined organic cause. To enhance measurement precision, improve comparability to other major studies, and detect potential differences in the importance of global vs. romantic attachment patterns, we assessed both concepts in our study.

1. Insecure attachment partially accounts for interindividual differences in the responses to acute pain, which are crucial for the transition to chronic pain [4]. Dysfunctional reactions to episodes of acute pain, which have been linked to insecure attachment, may be more important for the development of chronic pain without a clear organic cause compared to chronic pain with a clear organic cause. Based on empirical findings [20–22], we consider insecure attachment as a dispositional vulnerability to be of special relevance for the occurrence of pain conditions without clearly defined organic cause. Consequently, we expect insecure attachment patterns to be more frequently associated with medically unexplained musculoskeletal pain compared to pain from severe osteoarthritis.
2. Patients with insecure attachment patterns adapt to pain in more dysfunctional ways than securely attached patients [24]. Empirical findings indicate that this is also true for pain conditions with a clear somatic pathology [17, 28]. Therefore, we expect insecure attachment patterns to be associated with higher levels of symptom severity as measured by pain intensity, disability, and depression in both pain conditions. In addition, as the UMP group may comprise a different diagnostic entity due to the diagnosed mental health condition (somatoform disorder), which has the potential to impact results via multiple pathways [19], we used exploratory analyses to test differential effects of attachment by group interactions on these variables as well.

## Methods

### Participants

Two clinical samples were investigated prior to treatment in a cross-sectional design. Patients of both samples suffered from chronic musculoskeletal pain for a continuous period of at least six months. They presented themselves to the Department of Orthopedic Surgery of the University of Heidelberg between September 2008 and January 2010. At the time of admission, comprehensive medical and psychological diagnostic procedures were administered. To check whether pain intensity, pain location, or pain spreading was fully explained by specific somatic pathology, the results of a clinical examination were compared to diagnostic imaging by an orthopedic specialist. Furthermore, the Structured Diagnostic Interview for DSM-IV Axis I Disorders (SCID-I) was administered by a trained psychologist to detect mental disorders. Patients were included in the UMP group, if 1) they suffered from CWP [32], back pain, and/or limb pain in at least two pain sites; 2) pain intensity and/or spreading were not explained by diagnostic findings; 3) prior standard treatment consisting of at least one rehabilitation program or two inpatient treatments were ineffective; and 4) a somatoform disorder was diagnosed. Patients suffering from monolocular pain that was clearly explainable by severe osteoarthritis were included in the OA group. Patients with pain in other locations or with other somatic conditions were excluded from OA. Exclusion criteria for both samples included insufficient ability to communicate in German, age below 18 or over 80 years, and a diagnosis of psychosis, bipolar disorder, or neurological disorder.

**Fig 1:**
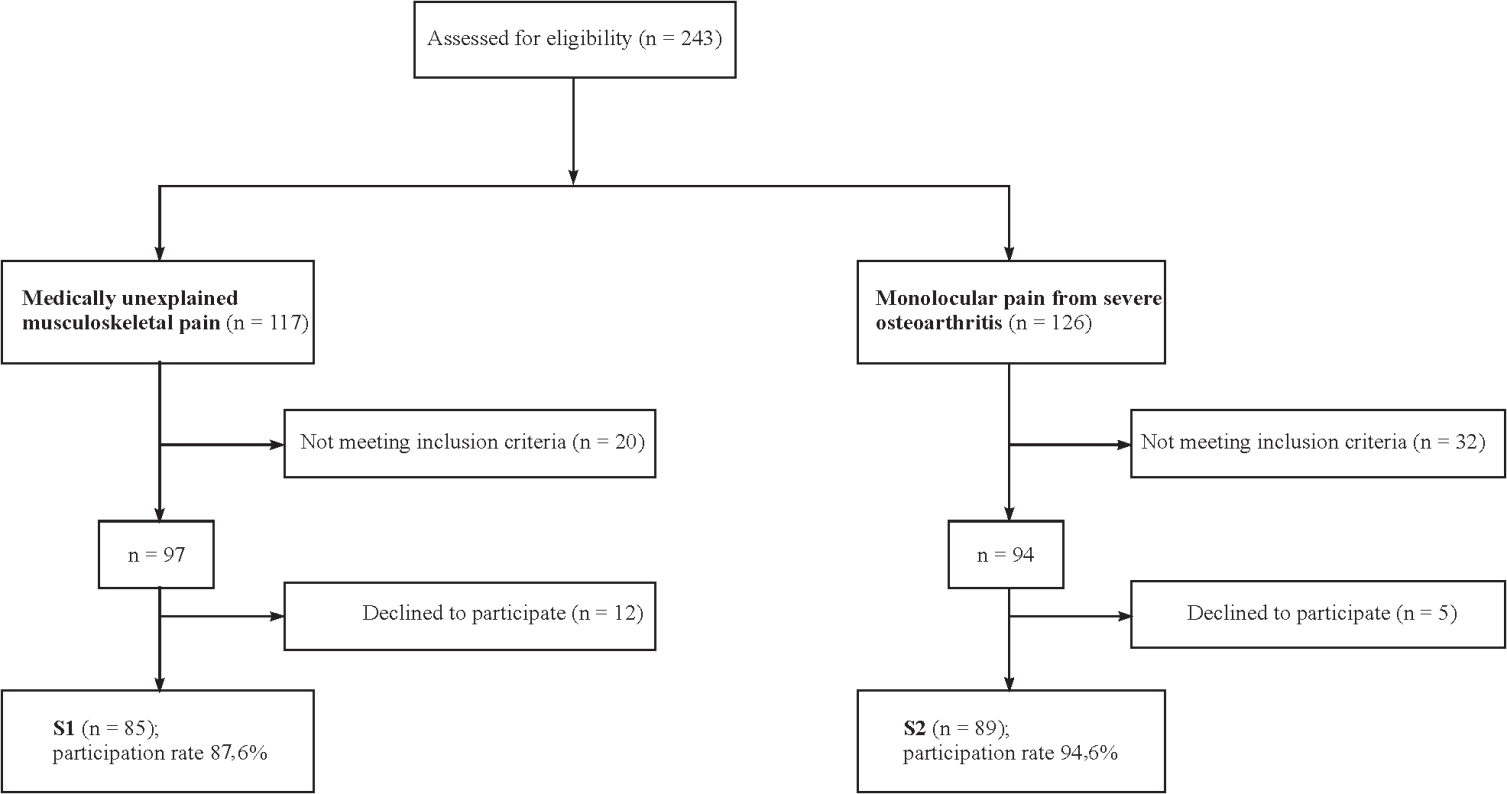
Flow chart of participant recruitment and attrition.

We calculated the necessary sample size for the entire sample (UMP and OA) with the G*Power Analysis software program for logistic regression analysis, and by reference to our primary hypothesis with regard to the secure-insecure RQ-2 attachment style categories. We expected a moderate effect of OR 3.0 for the association between insecure attachment with UMP (alpha = 0.05, two tailed). The results indicated that 172 participants were required. Of the 243 patients eligible (UMP: 117, OA: 126), 191 met the inclusion criteria (UMP: 97, OA: 94). Of these, 174 (91%) consented to participate in the study and completed the baseline questionnaires (UMP: 85, OA: 89) (Fig. 1). Data were acquired by means of standardized self-reported questionnaires that were personally administered and collected by the researchers on the first day of the patients’ outpatient or inpatient treatment (S1 Dataset). A structured interview for assessment of any psychiatric disorders was conducted within the first three days of treatment.

### Ethics statement

The study procedures were approved by the Institutional Review Board of the Medical Faculty, University of Heidelberg (S-220/2008), and complied with the ethical standards established in the Declaration of Helsinki [33]. Decisions of the Institutional Review Board of Heidelberg University also comply with the guidelines of ICH-GCP. Upon arrival at the department, the principal investigator stated the study purpose and procedures verbally to eligible patients. Additionally, all subjects were given written information about the study objectives and modalities (points of assessment, length of questionnaires), data preparation and pseudonymized data storage, the expected amount of commitment, the voluntary nature of participation, and their right to withdraw at any time. All participating patients provided written informed consent.

### Measures

#### Demographic information

Subjects were asked to report their age, gender, educational level, relationship and occupational status.

#### Psychopathology

The Structured Clinical Interview for DSM-IV Axis I Disorders (SCID-I) is a semi-structured interview for assessing DSM-IV Axis I diagnoses. It is considered diagnostic gold standard in psychiatric assessment, and has shown high reliability and superior validity when conducted by trained health professionals [34].

#### Assessment of adult attachment

As the human attachment system comprises representations of global models of the self and others in interaction and specific relationship experiences that are not perfectly correlated [35], we chose to administer two attachment measures—the Relationships Questionnaire and the Experiences in Close Relationships–Revised questionnaire—simultaneously.

The Relationships Questionnaire (RQ-2) is a brief self-report inventory that evaluates global attachment [10–11] according to Bartholomew’s four-category model of adult attachment. It consists of four short paragraphs, each describing one prototypical attachment pattern (e.g., the preoccupied prototype reads, “I want to be completely emotionally intimate with others, but I find that others are reluctant to get as close as I would like. I am uncomfortable being without close relationships, but I sometimes worry that others don’t value me as much as I value them”). Participants are asked to rate their agreement to each prototype on a 7-point Likert-type scale ranging from 1 (completely true) to 7 (completely false). From these four prototype ratings, the “model of self” and the “model of other” scales are calculated as proposed by the authors [10]. Hence, the RQ-2 provides categorical as well as dimensional information. Patients with positive models of both self and other were classified as “secure,” patients with negative/neutral models for both as “fearful,” and patients with mixed results as “preoccupied” or “dismissing.” If a patient was classified as neutral on both models, no general attachment style could be assigned, and the patient was excluded. The RQ-2 has an acceptable level of psychometric soundness for a brief screening instrument [16, 36], and it is relatively independent from self-deceptive biases [37]. In addition, it has been used in multiple international studies, and has proven to be an efficient screening instrument with good cross-cultural construct validity [38].

To assess romantic attachment, we used the German version [35] of the widely used, multi-item questionnaire Experiences in Close Relationships–Revised (ECR-R). The original version of the ECR-R [39] was developed within the framework of item response theory and covers the dimensions of attachment avoidance and attachment anxiety on two 18-item scales. In contrast to the general measurement of the RQ-2, the ECR-R specifically assesses attachment representations in romantic relationships (e.g., attachment anxiety: “I find that my partner(s) don’t want to get as close as I would like”; attachment avoidance: “I find it difficult to allow myself to depend on romantic partners”). The ECR-R is considered superior to conventional attachment questionnaires in terms of its psychometric properties [39], with high levels of test-retest stability, and convergent and discriminant validity [40].

#### Pain intensity and functional impairment

Subjective pain intensity over the preceding week was measured on a visual analogue scale (VAS) ranging from 0 (no pain) to 100 (worst imaginable pain). Each patient indicated a position on the VAS in response to the question “How severe was your pain on average in the last seven days?” The VAS is a valid and reliable instrument for measuring the intensity of pain [41].

Functional impairment was measured using the Hannover Dorsal Function Questionnaire (FFBH-R-R) [42], a questionnaire frequently used in Germany for the subjective assessment of physical function during daily life activities. The FFBH-R-R comprises 12 questions (e.g., “Are you able to wash and dry yourself from head to foot?” and “Are you able to sit on an un-upholstered chair for one hour?”), each with three possible answers: “Yes,” “Yes, with difficulty,” and “No.” The reliability, criterion-related validity, and sensitivity to changes of the FFBH-R-R have been described elsewhere [42]. Possible scores range from 0 to 100 and represent percentages of maximal functional capacity. We classified the results as follows: 80–100: normal; 60–80: moderate impairment; and <60: clinically relevant disability.

#### Depression

Psychological distress was measured by means of the depression subscale of the German version of the Hospital Anxiety and Depression Scale (HADS-D) [43], comprising seven items with responses on a 4-point scale. The HADS is widely used in medical settings and adequately assesses symptom severity and “caseness” of depression in somatic, psychiatric, and primary care patients, and in the general population [44].

### Data analysis

Descriptive statistics, such as measures of central tendency and of dispersion, were used for basic statistical analysis. Depending on data type, parametric t-tests, ANOVA, Pearson’s chi-square and Fisher’s exact test, respectively, were used for basic group comparisons between UMP and OA. Correlations between the RQ and ECR-R dimensions were assessed by means of Pearson’s r. To test the first hypothesis, odds ratios and confidence intervals from logistic regression models were used to quantify attachment styles in both groups. Separate linear regression models were constructed to examine the associations of global or romantic attachment with pain intensity, disability, and depression. In the logistic and linear regression models, independent variables were included in a forced-entry fashion. Before applying the regression analysis, the variables were tested for normal distribution via the Kolmogorov-Smirnov test, and graphical interpretation. All tests for significance were two-sided; p values below. 05 were considered statistically significant. Statistical analysis was performed using IBM SPSS Statistics 21.

Missing data resulted in decreased N values for regression analyses. Post hoc power calculation for logistic regression analysis with the given sample size of 148 patients with complete data for the examination of our first research question indicated a power of 80% with regard to the logistic regression analysis based on the secure-insecure categories, and of a power > 80 with regard to the analysis based on the four attachment style categories.

## Results

### Clinical and demographic characteristics

Descriptive statistics for demographic and clinical characteristics are shown in Table 1. Patients from the osteoarthritis sample were significantly older, better educated, and less depressed at baseline compared to patients with medically unexplained pain. However, the two groups did not differ significantly with regard to reported pain intensity in the last seven days or in their disability ratings, with the latter reaching clinically relevant levels in both groups.

**Table 1:** Demographic and clinical characteristics.

Characteristic	UMP	OA	p value ^a^
	unexplained musculoskeletal pain	pain from osteoarthritis	
	N = 85	N = 89	
Age (y) Mean (SD) range	48.9 (11,6) 20–76	58.0 (11,1) 19–77	.000**
Female sex % (n)	67.1 (57)	52.8 (47)	.055
Living in partnership % (n)	75.3 (64)	66.3 (59)	.192
College entrance % (n)	18.7 (14)	50.0 (42)	.000**
Employed % (n)	48.6 (35)	51.2 (43)	.748
Sick leave % (n)	12.5 (9)	4.8 (4)	.081
Pain last 7 days VAS Mean (SD)	60.3 (20,1)	60.8 (25,3)	.877
FFBH physical functioning Mean (SD)	55.5 (20.7)	48.0 (23,3)	.101
HADS depression Mean (SD)	8.0 (4.0)	4.2 (3,0)	.000**
RQ2 model of self Mean (SD)	2.7 (3.8)	4.8 (3,6)	.022*
RQ2 model of other Mean (SD)	0.4 (4.4)	1.7 (3,1)	.403
ECR-R attachment anxiety Mean (SD)	2.39 (1.04)	2.14 (1,02)	.116
ECR-R attachment avoidance Mean (SD)	2.41 (1.09)	2.19 (1,03)	.190

VAS, Visual Analogue Scale 0–100; FFBH, Hannover Dorsal Function Questionnaire; HADS, Hospital Anxiety and Depression Scale; RQ2 Relationships Questionnaire; ECR-R, Experiences in Close Relationships—Revised.

^a^ Calculated between samples by Pearson`s Chi^2^ (sex, partnership, college entrance), Fisher’s Exact (sick leave), t-test (age), ANOVA, controlled for gender, age, education (clinical characteristics).

* *p*<.05

** *p*<.01, two-sided.

The number and frequency of mental disorders diagnosed by means of the SCID-I are shown in Table 2. Within the UMP group, 46.3% met the criteria for CWP [32], 33.7% suffered from back and limb pain, and 20% from back pain or limb pain. In accordance with the inclusion criteria, all individuals in the UMP condition suffered from a somatoform disorder (89.8% somatoform pain disorder; 10.2% somatization disorder), with half of them (50.6%) diagnosed with at least one psychiatric comorbidity (38.8% with one additional disorder; 10.6% with two or more additional disorders). In contrast, 60.7% of the OA patients were not diagnosed with any psychiatric disorder, with only 6.7% of patients having two diagnoses.

**Table 2:** Number and frequencies of SCID-I-Diagnoses according to DSM-IV.

	UMP	OA
	medically unexplained musculoskeletal pain	pain from osteoarthritis
	N = 85	N = 89
Number of mental disorders	% (N)	% (N)
0	0 (0)	60.7 (54)
1	49.4 (42)	32.6 (29)
2	38.8 (33)	6.7 (6)
3	10.6 (9)	-
4	1.2 (1)	-
Frequencies of mental disorders	% (N)	% (N)
Somatoform disorders	100 (85)	0 (0)
Depressive disorders	21.2 (18)	10.1 (9)
Anxiety-/ obs.-compulsive disorders	28.2 (24)	34.8 (31)
Eating disorders	14.1 (12)	1.1 (1)

SCID-I, Structured Diagnostic Interview for DSM-IV Axis I Disorders.

### Global and romantic attachment

**Fig 2:**
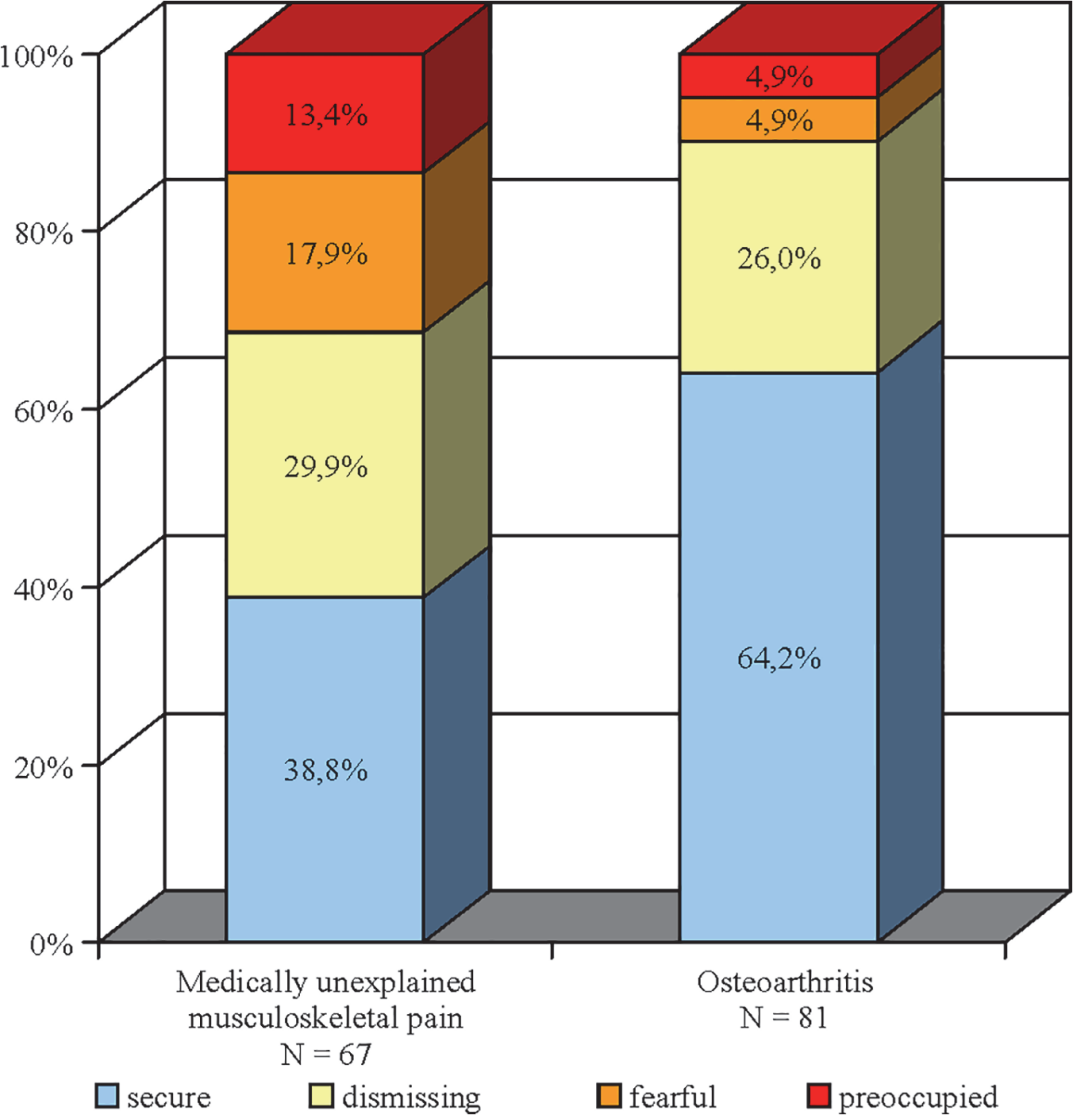
Global attachment styles in medically unexplained musculoskeletal pain and osteoarthritis. Global attachment styles (one “secure” and three insecure: “preoccupied”, “dismissing”, and “fearful”) were derived from the Relationships Questionnaire (RQ-2), a brief self-report inventory that evaluates global attachment according to Bartholomew’s four-category model of adult attachment.

The frequencies of global RQ attachment style categories are presented in Table 3, the proportions are displayed in Fig. 2. Pain conditions differed significantly according to attachment security (chi square test, χ^2^ (1) = 11,24, p = .002): about one-third of UMP patients were classified as securely attached (38.8%), while this was true for two-thirds (64.2%) of OA patients. Preoccupied and fearful attachment styles were characterized by a negative model of self. The increased proportion of these categories is also reflected in lower dimensional RQ _model of self_ scores in UMP compared to OA. In contrast, levels of the romantic ECR-R dimensions did not differ between the groups (Table 1). RQ scores for global attachment and the corresponding ECR-R dimensions for romantic attachment were moderately correlated, r (152) = -0.529, p <. 001 for RQ _model of self_ and ECR-R attachment anxiety, and r (152) = 0.433, p <. 001 for RQ _model of other_ and ECR-R attachment avoidance.

**Table 3:** RQ attachment style frequencies and Odd’s ratios.

Attachment Style	UMP	OA	Odds ratio for UMP
	Medically unexplained musculoskeletal pain	pain from osteoarthritis	
	N = 67	N = 81	
	N	N	OR^a^ (95% CI)
Secure	26	52	Ref
Insecure total	41	29	3,39 (1.44–8.01) * *
Dismissing	20	21	1.87 (0.69–5.05)
Preoccupied	12	4	7.94 (1.76–35.95)* *
Fearful	9	4	8.02 (1.66–38.80)*

RQ: Relationships Questionnaire.

^a^ OR for UMP, reference OA, controlled for gender, age, education, relationship status, pain intensity.

* *p*<.05

**p<.01, two-sided.

### Associations between attachment and pain condition

In order to examine the hypothesis that insecure attachment is more frequently associated with UMP compared to OA, we conducted logistic regression analyses with pain condition as the dependent variable. We controlled for gender, age, education, relationship status, and pain intensity, because the severity of pain may augment insecure attachment [45]. In addition to age, educational level, and relationship status, global attachment also significantly predicted pain condition; patients with an insecure attachment style were 3.4 times (95% CI: 1.44 to 8.01, p = .005) more likely to suffer from UMP than from OA, compared to patients with secure attachment, reflecting a moderately large effect size (d = 0.67). This was particularly true for patients with a preoccupied (OR 7.94, 95% CI: 1.76 to 35.95, p = .007), and a fearful attachment pattern (OR 8.02, 95% CI: 1.66 to 38.80, p = .01) (Table 3). To examine the stability of our results in a larger number of cases, we conducted a further logistic regression analysis with the RQ _model of self_ and RQ _model of other_ dimensions as independent variables. Age, educational level, relationship status, and RQ _model of self_ (OR 0.89, referring to the described 25-point model of self-scale) were significant predictors (Table 4), indicating that patients with a negative model of self were more likely to suffer from UMP. In contrast, romantic attachment measured by the ECR-R was not more frequently associated with UMP; neither attachment anxiety nor attachment avoidance predicted pain condition (Table 4).

**Table 4:** Attachment dimensions and pain condition.

	Attachment domain			
	Global attachment^a^		Romantic attachment^a^	
Predictors	OR	95% CI	OR	(95% CI)
Age	0.94**	[0.91, 0.97]	0.93**	[0.90, 0.97]
sex	0.61	[0.28, 1.35]	0.62	[0.28, 1.38]
Living in partnership	5.19**	[1.88, 14.33]	5.33**	[1.86, 15.36]
Education (college entrance)	0.27**	[0.12, 0.61]	0.22**	[0.09, 0.50]
Pain intensity VAS	1.00	[0.98, 1.01]	0.99	[0.97, 1.01]
RQ model of self	0.89*	[0.79, 0.99]		
RQ model of other	0.93	[0.84, 1.04]		
ECR-R attachment anxiety	-		1.28	[0.80, 2.03]
ECR-R attachment avoidance	-		1.07	[0.69–1.65]

N = 152; OR = odds ratio; CI = confidence interval.

VAS, Visual Analogue Scale 0–100; RQ Relationships Questionnaire, RQ model of self / model of other -12–+12.

ECR-R, Experiences in Close Relationships—Revised, ECR-R attachment anxiety/attachment avoidance 1–7.

^a^ Variables entered simultaneously.

* *p*<.05

** *p*<.01, two-sided.

### Associations between attachment and symptom severity

To test our hypothesis that insecurely attached patients in both pain conditions will report higher levels symptom severity (pain intensity, disability, and depression), hierarchical multiple regression analyses were conducted separately for each attachment measure (global and romantic). We controlled for gender, age, education, and relationship status. Moreover, the clinical characteristics not predicted by the model were entered as covariates due to the well-known interaction between depression, pain perception, and disability in chronic pain patients [1–2]. In the first block, these demographic and clinical control variables were force entered. The second block consisted of the single variable *pain condition* (OA/UMP). *Global attachment* (RQ _model of self_ /RQ _model of other_) and *romantic attachment* (ECR-R attachment anxiety/ECR-R attachment avoidance) were entered in the third block, respectively. In the fourth block, the *interaction* between pain condition and global attachment, or romantic attachment, were entered. Standardized beta regression coefficients for the predictors (except for the control variables) and R^2^ values of the regression models are shown in Tables 5–7.

**Table 5:** Regression analyses predicting pain intensity from pain condition, attachment dimensions, and interaction between pain condition and attachment dimensions.

	Attachment domain			
	Global		Romantic	
Predictor	Δ*R*²	β	Δ*R*²	β
Step 1	.08		.08	
Control variables^a^				
Step 2	.01		.00	
Pain condition^b^		-.05		. 33
Step 3^c^	.01		.01	
RQ model of self		.22		
RQ model of other		-.03		
ECR-R attachment auxiety				. 82*
ECR-R attachment avoidance				-.14
Step 4	.00		.05	
Pain condition x RQ model of self		-.15		
Pain condition x RQ model of other		.14		
Pain condition x ECR-R attachment anxiety				-1.04*
Pain condition x ECR-R attachment avoidance				. 27
Total *R* ^2^	.10		.14	
n	151		145	

^a^ Control variables included gender, age, education, relationship status, disability, and depression.

^b^ Pain condition: UMP/OA.

^c^ RQ Relationships Questionnaire, RQ model of self / model of other -12–+ 12; ECR-R, Experiences in Close Relationships—Revised, ECR-R attachment anxiety/attachment avoidance 1–7.

* *p*<.05

**p<.01, two-sided.

**Table 6:** Regression analyses predicting disability from pain condition, attachment dimensions, and interaction between pain condition and attachment dimensions.

	Attachment domain			
	Global		Romantic	
Predictor	Δ*R*²	β	Δ*R*²	β
Step 1	.05		.04	
Control variables				
Step 2	.02		.02	
Pain condition		.24		.24
Step 3	.01		.04	
RQ model of self		.05		
RQ model of other		.13		
ECR-R attachment anxiety				-.28
ECR-R attachment avoidance				.51
Step 4	.00		.01	
Pain condition x RQ model of self		-.11		
Pain condition x RQ model of other		-.03		
Pain condition x ECR-R attachment anxiety				.31
Pain condition x ECR-R attachment avoidance				.39
Total *R* ^2^	.08		.10	
n	151		145	

^a^ Control variables included gender, age, education, relationship status, pain intensity, and depression.

^b^ Pain condition: UMP/OA.

^c^ RQ Relationships Questionnaire, RQ model of self / model of other -12–+ 12; ECR-R, Experiences in Close Relationships—Revised, ECR-R attachment anxiety/attachment avoidance 1–7.

* *p*<.05

**p<.01, two-sided.

**Table 7:** Regression analyses predicting depression from pain condition, attachment dimensions, and interaction between pain condition and attachment dimensions.

	Attachment domain			
	Global		Romantic	
Predictor	Δ*R*²	β	Δ*R*²	β
Step 1	.07		.08	
Control variables				
Step 2	.19		.20	
Pain condition		. 40**		.48**
Step 3	.15		.17	
RQ model of self		-.45*		
RQ model of other		.11		
ECR-R attachment anxiety				. 69**
ECR-R attachment avoidance				-.19
Step 4	.01		.02	
Pain condition x RQ model of self		.08		
Pain condition x RQ model of other		-.24		
Pain condition x ECR-R attachment anxiety				. 08
Pain condition x ECR-R attachment avoidance				-.24
Total *R* ^2^	.41		.46	
n	151		145	

^a^ Control variables included gender, age, education, relationship status, pain intensity, and disability.

^b^ Pain condition: UMP/OA.

^c^ RQ Relationships Questionnaire, RQ model of self / model of other -12–+ 12; ECR-R, Experiences in Close Relationships—Revised, ECR-R attachment anxiety/attachment avoidance 1–7.

* *p*<.05

**p<.01, two-sided.

#### Global attachment and symptom severity

Neither global insecure attachment nor pain condition or their interaction predicted pain intensity (s. Table 5). Only two control variables significantly (or by tendency) accounted for the variance of pain intensity: patients with a lower educational level (B = -7.91, 95% CI: -15.97 to 0.16, β = -0.17, p = .055) and lower physical functioning (B = -0.20, 95% CI: -0.37 to -0.03, β = -0.19, p = .022) were more likely to report higher pain severity. In the model assessing predictors of disability, no significant associations were found (Table 6). In contrast, the UMP condition (B = 3.04, 95% CI: 1.47 to 4.61, β = 0.40, p = .000) and lower RQ _model of self_ (B = -0.45, 95% CI: -0.70 to -0.02, β = -0.48, p = .042) were significantly related to depressive symptoms (s. Table 7). Furthermore, the control variables female sex (B = 1.56, 95% CI: 0.52 to 2.61, β = 0.20, p = .004) and pain intensity (B = 3.04, 95% CI: 1.47 to 4.61, β = 0.15, p = .034) were associated with depression. Global attachment accounted for 15% of the variance in depression, reflecting a moderate effect. Overall, this regression model explained 41% of the variance ((F [11,151] = 8.59, p <. 0001).

#### Romantic attachment and symptom severity

Higher levels of ECR-R attachment anxiety were significantly associated with pain intensity (B = 18.12, 95% CI: 4.27 to 31.97, β = 0.82, p = .011). There was also a significant interaction between attachment anxiety and pain condition for pain intensity (B = -11.34, 95% CI: -20.11 to -2.57, β = -1.04, p = .012) (s. Table 5). In other words, romantic attachment anxiety was associated with higher pain intensity, and this relationship was more pronounced in the OA group compared to the UMP group. The control variables education (B = -8.73, 95% CI: -16.89 to -0.58, β = -0.18, p = .036) and physical functioning (B = -0.20, 95% CI: -0.37 to -0.03, β = -0.19, p = .024) were also significant. However, this regression model accounted for only 14% of the variance in pain intensity (F [11,145] = 1.96, p = .037). Romantic attachment and interaction between romantic attachment and pain condition together explained 6% (Table 5), reflecting a small effect. In the regression model for disability, no significant predictors were found (Table 6). However, in the model assessing predictors for depression, having UMP (B = 3.70, 95% CI: 1.05 to 6.35, β = 0.48, p = .007), higher attachment anxiety scores (B = 2.52, 95% CI: 0.70 to 4.35, β = 0.69, p = .007), and being female (B = 1.22, 95% CI: 0.70 to 2.28, β = 0.15, p = .024) was associated with higher depression scores. No significant interactions were found. Overall, our regression model explained 46% of the total variance in depression (F [11,145] = 8.79, p <. 0001), while romantic attachment accounted for 17% of the variance.

## Discussion

The present study sought to inform our understanding of insecure attachment as a dispositional vulnerability for the occurrence and severity of chronic pain. We investigated two well-defined, adequately large samples of patients with and without a clear somatic pathology. This is the first study to use two measures assessing different attachment domains in chronic pain. The investigation of pain syndromes with different etiologies, and the combination of attachment measures allowed us to detect differences between pain conditions and types of attachment representations, helping to clarify inconsistencies in previous research.

### Associations between attachment and pain condition

We hypothesized that insecure attachment patterns are more frequently associated with medically unexplained musculoskeletal pain, compared to pain from severe osteoarthritis. This was confirmed for global attachment representations, but not for romantic attachment domains. There was a higher prevalence of global insecure attachment styles in UMP (nearly 2/3), whereas the proportion of 36% global insecure attachment styles in osteoarthritis matches the proportion found in a large nationally representative sample of adults from the United States [46]. Age, education, relationship status, and importantly, global insecure attachment, were significant predictors of pain condition. Patients with global insecure attachment styles were 3.4 times more likely to suffer from UMP than OA compared to patients with secure attachment. Furthermore, UMP was strongly associated with the preoccupied and fearful subtypes of global insecure attachment, whereas the proportion of dismissing attachment did not differ between pain conditions. Analyses on the global attachment dimensions supported these findings; preoccupied and fearful attachment styles were defined by a negative model of self, which was negatively associated with UMP. Our results indicated that the experience of UMP, but not OA, is associated with global preoccupied and fearful attachment. Due to the cross-sectional study design, we are not able to draw conclusions on the direction of this relationship. One possible interpretation is that subjects who are generally anxious about the responsiveness of others (high global attachment anxiety or fearful and preoccupied style using the categories of Bartholomew and Horowitz [11]) are at increased risk of developing chronic pain following episodes of pain without clear pathology. This interpretation is in accordance with studies linking maladaptive coping strategies for acute pain episodes mainly with preoccupied and fearful attachment or high attachment anxiety [5, 15, 23, 30]. The unequal proportion of global insecure attachment styles, i.e., the different models of self between the pain syndromes, may be partly an effect of a recruitment bias resulting from the influence of attachment style on help-seeking behavior [17, 24, 47]. However, our results are consistent with Davies et al.’s findings that preoccupied attachment is most strongly associated with CWP in a population-based sample [29].

Interestingly, romantic attachment insecurity was not directly related to medically unexplained pain. Romantic attachment anxiety and romantic attachment avoidance did not differ between pain conditions and were similar to those of nonclinical samples [35]. These divergent results reflect only moderate statistical overlap between RQ-2 scores and their corresponding ECR-R dimensions found in this study, similar to the correlations reported in other studies [35, 40]. Although some of the observed differences between RQ-2 and ECR-R results may be related to measurement error due to differences in precision between the instruments, they could also be attributable to their different relational foci [12–13]. This is consistent with attachment theory and research that propose that attachment representations are likely to differ between relationship domains [12], and that insecure attachment orientations may shift to more secure romantic representations as a result of positive experiences in intimate relationships [48]. Our findings indicate that UMP is associated with insecure attachment in general, as it affects a variety of interactional contexts (e.g., provider-patient-relationships), but not stringently with insecure attachment in romantic love. Global attachment insecurity was overrepresented in UMP, but need not necessarily be accompanied by attachment insecurity in intimate relationships.

### Associations between attachment and symptom severity

We also expected attachment insecurity to be associated with pain intensity, disability, and depression in both pain conditions. Our predictions were confirmed with regard to pain and depression, but not disability. Pain intensity and functional impairment were independent of global attachment, across secure/insecure categories, and model of self/other dimensions. These results support earlier findings [25–27], suggesting that pain intensity and disability are not directly associated with global attachment patterns. In contrast, an association between romantic attachment anxiety and pain intensity emerged, that was more pronounced in the osteoarthritis group. This is in line with some studies that used measures referring to romantic love in patients suffering from medical explained pain [17, 28]. Our findings indicate that social support from intimate partners is particularly important in the pain experience. Recent research accentuated the importance of romantic attachment anxiety on perceived social support and pain perception [15, 49], but also healthcare utilization in other medical conditions [50]. However, it is unclear why the association between pain intensity and attachment anxiety was more pronounced in OA compared to UMP, and why the contribution to the variance of pain intensity was rather low. Other variables may be of importance, for example limited accessibility of a romantic partner for providing social support, or anxiety of loss of a partner due to possible adverse health conditions in the generally older OA group. The stability of this finding thus requires replication and further investigation of different types of relationships in different pain conditions.

Regarding depression, consistent associations were found in both groups and measures. Lower global attachment model of self-scores were associated with higher depression in both pain conditions. Similar results were obtained using the romantic attachment measure. Patients with higher romantic attachment anxiety were likely to report higher psychological distress in terms of depression. The presence of a somatoform disorder as a mental health condition in the UMP group yielded no effects of attachment by group interactions on depression. While associations between adult attachment and depression have been documented in other samples [51–52], our study shows that global and romantic attachment patterns are associated with depression in the context of pain, independent of underlying organic pathology. Furthermore, this relationship seemed to be attributable mainly to the attachment anxiety (or model of self) dimension. These findings are consistent with those of previous studies, where particularly fearful and preoccupied attachment styles (characterized by a negative model of self or high attachment anxiety), were linked to lower self-esteem and increased focus on negative affect in general [7, 47], and to lower self-efficacy and depression in the context of pain [26, 31]. Although it was not the main focus of this study, it should be noted that we found markedly increased depression scores in the UMP group. Although Berkson’s bias cannot be ruled out, the restrictive inclusion criteria and the relatively low rate of expert-rated depressive disorders (21%) in the UMP group argue against a distinct referral bias [53].

### Limitations

Our results must be regarded as preliminary due to some methodological limitations. First, owing to the cross-sectional nature of our study, it remains unclear whether the experience of UMP influences the activation of fearful or preoccupied attachment [54], or whether the associations between these global attachment styles and UMP reflect a specific, pre-existing vulnerability in this subgroup. This limitation is also relevant to the associations between attachment and symptom severity. For example, the relationship between attachment insecurity and pain is likely to be bidirectional, for the experience of pain may augment attachment anxiety [45]. Longitudinal data are therefore needed to elucidate the direction of the relationship between insecure attachment and the onset and severity of chronic pain. Second, it was not possible to compare demographic, clinical, or attachment variables between the participants and patients who refused to take part in the study. However, personally addressing all potential study participants generated a very high response rate (91%), which argues against a meaningful distortion of the results by sample self-selection. Third, general attachment styles, in contrast to romantic attachment, were assessed by a four-item instrument with moderate reliability [55]. The RQ-2 was nevertheless applied because it has been implemented in multiple studies and considered an efficient screening instrument with good cross-cultural construct validity [38]. Furthermore, the RQ-2 is relatively independent from social desirability biases [37]. Fourth, we found unexpectedly low frequencies of preoccupied and fearful attachment styles in the osteoarthritis sample, resulting in small cell sizes in RQ-2 categories. On account of this, we replicated the calculation with the help of the dimensional RQ-2 ratings. Finally, attachment was assessed exclusively by self-rating measures. Thus, potentially important unconscious manifestations of the attachment system could not be covered [56]. Using sophisticated interviewing techniques to diagnose adult attachment might significantly increase the validity of future results.

## Summary and Conclusions

Overall, the results supported our prediction that insecure attachment is more prevalent in medically unexplained pain compared to pain with a clear organic cause, but that poorer outcome in chronic pain relates to insecure attachment independently of organic pathology. As described above, our study revealed substantial differences in the relationships of global vs. romantic attachment patterns with the occurrence and outcome of chronic pain. First, medically unexplained pain was associated with global attachment insecurity, but not with romantic attachment insecurity. Second, pain intensity was linked to romantic attachment anxiety, but not to global attachment insecurity. These differences between the levels of attachment representation in chronic pain have implications for the development of attachment-informed strategies in prevention and treatment. Therefore, our results underline the need to integrate generic and relation-specific attachment representations in research approaches to increase the understanding of their differential importance in the context of chronic pain.

Our results tentatively suggest the importance of incorporating attachment-informed interventions into the prevention and rehabilitation of chronic pain. The finding that general patterns of insecure attachment are associated with UMP requires early intervention strategies that consider the specific interactional needs that are linked to these attachment styles. For example, patients with a preoccupied attachment style may be impaired in their ability to benefit from self-management-focused treatment strategies, and may need more reassuring, supporting approaches to gradually foster their self-efficacy and independence, whereas patients with a fearful attachment style may need encouragement towards self-disclosure and appropriate help-seeking behavior. Our findings argue against a direct impact of global attachment patterns on the severity of pain and disability. However, they confirm earlier observed associations of insecure romantic attachment and pain intensity, suggesting the importance of involving close others in treatments to reduce the experience of pain with and without clearly identifiable pathology. As global and romantic attachment insecurity were linked to the severity of depressive symptoms in both unexplained musculoskeletal pain and osteoarthritis, rehabilitation programs for chronic pain syndromes should consider not only depression but also different forms of attachment insecurity by implementing relationship-focused approaches.

## Supporting Information

S1 Dataset SPSS data file showing attachment, pain, disability, and depression. This data file contains raw data from questionnaires measuring attachment, pain, disability and depression in two different pain conditions. All regression analyses are based on these data. The file includes pain condition (pain_condition), age (Age), sex (Sex), living in partnership (Partnerschaft_kat), college entrance (Education), employment (Employment), sick leave (EM_Rente_jn), pain intensity last seven days (Schmerz_1_t1), raw data from HADS, FFbH, ECR-R, RQ, global attachment dimensions (RQ_t1_modelself, RQ_t1_modelother), global attachment styles (RQ_4Bindungsstile).
(SAV)
